# Supplementary material for: Modelling approaches for estimating vaccine effectiveness of consecutive SARS-CoV-2 variant sublineages in the absence of study-specific genetic sequencing data, VEBIS hospital network, Europe, 2023/24
Source: PLoS One. 2026 Mar 9;21(3):e0343988. doi: 10.1371/journal.pone.0343988 (PMC12970855; doi:10.1371/journal.pone.0343988)
Supplement: S1 Section — (PDF) [file pone.0343988.s002.pdf]

S1 Section. Description of the proxy determination for XBB and BA.2.86 predominance periods

The study periods were restricted to the weeks where the consecutive variant sublineages (VSLs) of interest were predominant. We defined weekly thresholds for both XBB and BA.2.86 VSL predominant periods as ≥ 60%, by country (Figure S1). We excluded weeks with a low number of sequenced samples (n<17) to determine the start/end of the predominance period and, for countries with low quality data, used data from neighbouring countries, in addition to their own data (summed across numerator and denominator), to produce the final variant proportion time series (S1 Figure). The minimum number of sequenced samples (17) was determined based on the coefficient of variation (CV) for the proportion, given by CV = $\frac{\sqrt{\frac{\hat{p}(1-\hat{p})}{n}}}{\hat{p}}$. We assumed CV=0.2, for a proportion of 0.6 and found the minimum sample size (n).
