# Supplementary material for: Modelling approaches for estimating vaccine effectiveness of consecutive SARS-CoV-2 variant sublineages in the absence of study-specific genetic sequencing data, VEBIS hospital network, Europe, 2023/24
Source: PLoS One. 2026 Mar 9;21(3):e0343988. doi: 10.1371/journal.pone.0343988 (PMC12970855; doi:10.1371/journal.pone.0343988)
Supplement: S2 Section — (PDF) [file pone.0343988.s003.pdf]

S2 Section. Description of the estimation of the weekly BA.2.86 VSL proportion

The variant proportion data was sourced from ERVISS Github [1].

The model equation used to estimate the weekly time series for each country is presented in Eq. S1, where p is the reported proportion of BA.2.86 (either from the country or, when missing data/low number of sequenced samples, based on the country and its neighbours together), and t represents time in weeks.

$$\log\left(\frac{p}{1-p}\right) = \beta_0 + \beta_1 t \quad \text{(Eq. S1)}$$
