# Supplementary material for: Modelling approaches for estimating vaccine effectiveness of consecutive SARS-CoV-2 variant sublineages in the absence of study-specific genetic sequencing data, VEBIS hospital network, Europe, 2023/24
Source: PLoS One. 2026 Mar 9;21(3):e0343988. doi: 10.1371/journal.pone.0343988 (PMC12970855; doi:10.1371/journal.pone.0343988)

S1 Fig. Weekly BA.2.86 VSL proportion reported by the country, the proportion calculated using neighbouring countries and the final estimated time series using logistic regression used in the category and proportion models, based on TESSy/GISAID data (ECDC ERVISS Github), VEBIS hospital network, Europe.

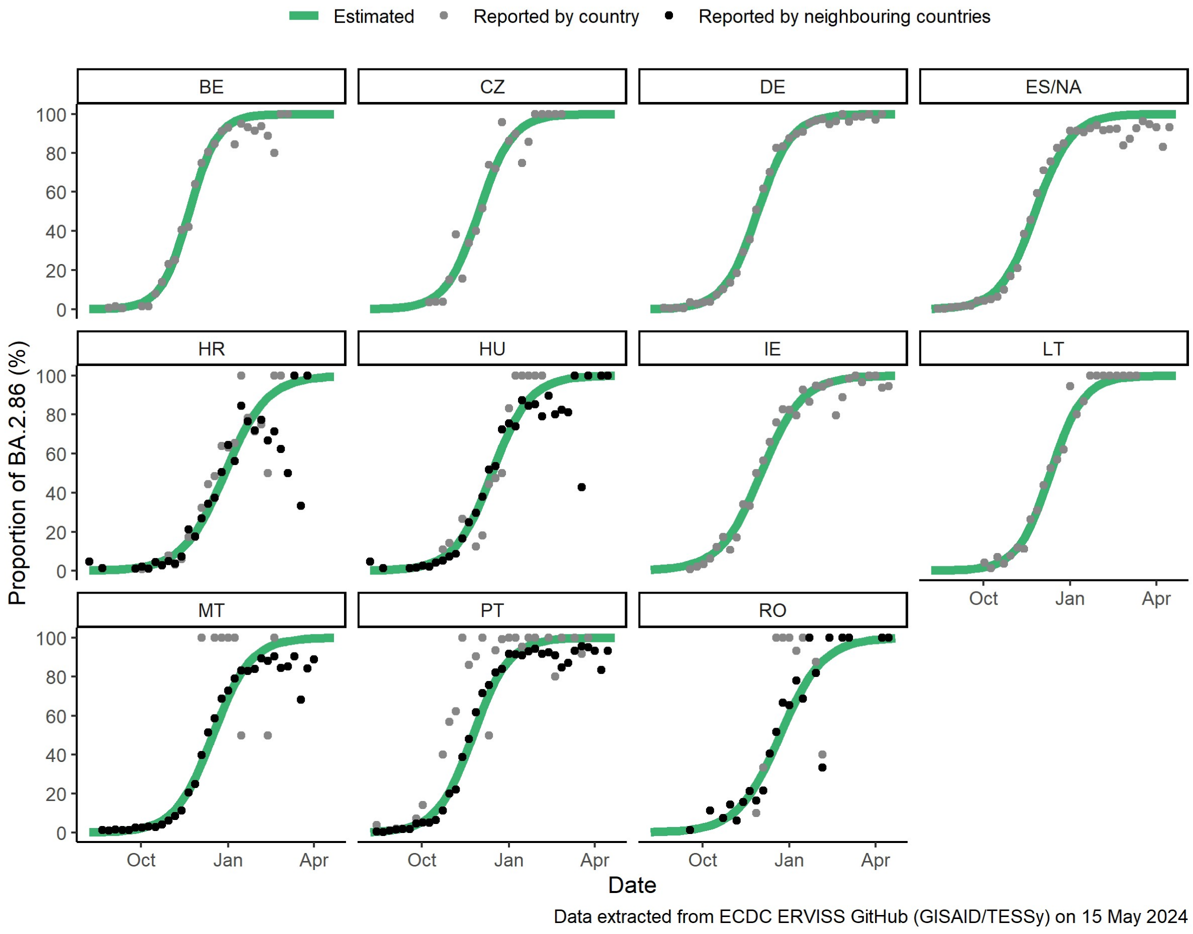

Supplement: S1 Fig — (PDF) [file pone.0343988.s004.pdf]
