# Supplementary material for: Modelling approaches for estimating vaccine effectiveness of consecutive SARS-CoV-2 variant sublineages in the absence of study-specific genetic sequencing data, VEBIS hospital network, Europe, 2023/24
Source: PLoS One. 2026 Mar 9;21(3):e0343988. doi: 10.1371/journal.pone.0343988 (PMC12970855; doi:10.1371/journal.pone.0343988)
Supplement: S2 Table — (PDF) [file pone.0343988.s005.pdf]

S2 Table. Start date of the 2023/24 vaccination campaign by site, VEBIS hospital study, Europe.

Site	Date vaccination campaign started	XBB.1.5 vaccine introduction
Belgium (BE)	15 Sep 2023	15 Sep 2023
Czechia (CZ)	04 Aug 2023	04 Aug 2023
Germany (DE)	18 Sep 2023	18 Sep 2023
Spain (ES)	25 Sep 2023	25 Sep 2023
Croatia (HR)	18 Sep 2023	18 Sep 2023
Hungary (HU)	01 Oct 2023	01 Dec 2023
Ireland (IE)	02 Oct 2023	02 Oct 2023
Lithuania (LT)	05 Oct 2023	05 Oct 2023
Malta (MT)	09 Oct 2023	09 Oct 2023
Navarre region, Spain (NA)[a]	16 Oct 2023	16 Oct 2023
Portugal (PT)	29 Sep 2023	29 Sep 2023
Romania (RO)	02 Oct 2023	17 Jan 2024

[a] The 2023 autumn vaccination campaign started earlier in the Navarre region in Spain (25 September 2023), with sparse administration of vaccines. Vaccines started being administered in larger quantities after 16 October 2023.
