# Supplementary material for: Modelling approaches for estimating vaccine effectiveness of consecutive SARS-CoV-2 variant sublineages in the absence of study-specific genetic sequencing data, VEBIS hospital network, Europe, 2023/24
Source: PLoS One. 2026 Mar 9;21(3):e0343988. doi: 10.1371/journal.pone.0343988 (PMC12970855; doi:10.1371/journal.pone.0343988)
Supplement: S3 Table — (PDF) [file pone.0343988.s006.pdf]

S3 Table. Start date[a] of the XBB and BA.2.86 VSL predominant periods for a 60% predominance threshold,[b] by site, VEBIS hospital study, Europe

Site	BA.2.86<br>First date in start week	XBB<br>First date in start week	XBB<br>Last date in end week
Belgium (BE)	27 Nov 2023	27 Feb 2023	12 Nov 2023
Czechia (CZ)	11 Dec 2023	06 Feb 2023	26 Nov 2023
Germany (DE)	04 Dec 2023	06 Mar 2023	26 Nov 2023
Spain (ES)	04 Dec 2023	27 Feb 2023	12 Nov 2023
Croatia (HR)	01 Jan 2024[c]	27 Feb 2023	10 Dec 2023
Hungary (HU)	25 Dec 2023[c]	27 Mar 2023[c]	10 Dec 2023[c]
Ireland (IE)	11 Dec 2023	13 Feb 2023	26 Nov 2023
Lithuania (LT)	25 Dec 2023	20 Mar 2023	03 Dec 2023
Malta (MT)	25 Dec 2023[c]	13 Mar 2023[c]	03 Dec 2023[c]
Portugal (PT)	27 Nov 2023[c]	13 Mar 2023	15 Oct 2023
Romania (RO)	01 Jan 2024[c]	06 Mar 2023[c]	17 Dec 2023[c]

AT: Austria; BG: Bulgaria; EL: Greece; ES: Spain; HR: Croatia; HU: Hungary; IT: Italy; MT: Malta; PT: Portugal; RO: Romania; SI: Slovenia; SK: Slovakia; VEBIS: Vaccine Effectiveness, Burden and Impact Studies; VSL: variant/sublineage.

[a] Date of first day of the start week.

[b] Data on SARS-CoV-2 circulation available from ECDC ERVISS Github, extracted on 16 May 2024.

[c] Start date week was calculated based on data from the site country and its neighbouring countries together: HR (neighbours = HU, SI); HU (neighbours = AT, HR, RO, SI, SK); MT (neighbours = HR, EL, IT); PT (neighbours = ES); RO (neighbours = BG, HU).
