# Supplementary material for: Modelling approaches for estimating vaccine effectiveness of consecutive SARS-CoV-2 variant sublineages in the absence of study-specific genetic sequencing data, VEBIS hospital network, Europe, 2023/24
Source: PLoS One. 2026 Mar 9;21(3):e0343988. doi: 10.1371/journal.pone.0343988 (PMC12970855; doi:10.1371/journal.pone.0343988)
Supplement: S3 Section — (PDF) [file pone.0343988.s007.pdf]

S3 Section. Demonstration of ratio of odds ratios (ORs) in case–case studies

S4 Table. Example of a table with the number of XBB and BA.2.86 cases and controls by their vaccination status, VEBIS hospital study, Europe

Vaccination status	XBB cases	BA.2.86 cases	Controls
Vaccinated	a	b	c
Unvaccinated	d	e	f

In case–case studies, the odds of vaccination between BA.2.86 and XBB VSLs ($OR_{BA.2.86 \quad vs \quad XBB}$) is given by Eq. S2:

$$OR_{BA.2.86 \quad vs \quad XBB} = \frac{bd}{ea} \quad \text{(Eq. S2)}$$

The ratio of the OR between the two variants ($\frac{OR_{BA.2.86}}{OR_{XBB}}$) is given by Eq. S3, which amounts to the same in Eq. S2:

$$\frac{OR_{BA.2.86}}{OR_{XBB}} = \frac{\frac{bf}{ce}}{\frac{af}{cd}} = \frac{bd}{ea} \quad \text{(Eq. S3)}$$

where the OR of vaccination against XBB (OR_{XBB}) and BA.2.86 ($OR_{BA.2.86}$) VSLs are given by:

$$OR_{XBB} = \frac{af}{cd} \quad \text{(Eq. S4)}$$

$$OR_{BA.2.86} = \frac{bf}{ce} \quad \text{(Eq. S5)}$$
