# Supplementary material for: Modelling approaches for estimating vaccine effectiveness of consecutive SARS-CoV-2 variant sublineages in the absence of study-specific genetic sequencing data, VEBIS hospital network, Europe, 2023/24
Source: PLoS One. 2026 Mar 9;21(3):e0343988. doi: 10.1371/journal.pone.0343988 (PMC12970855; doi:10.1371/journal.pone.0343988)
Supplement: S3 Fig — (PDF) [file pone.0343988.s009.pdf]

S3 Fig. Patient exclusion flowchart for the BA.2.86 sequenced data (SD) analysis, VEBIS hospital study, Europe.

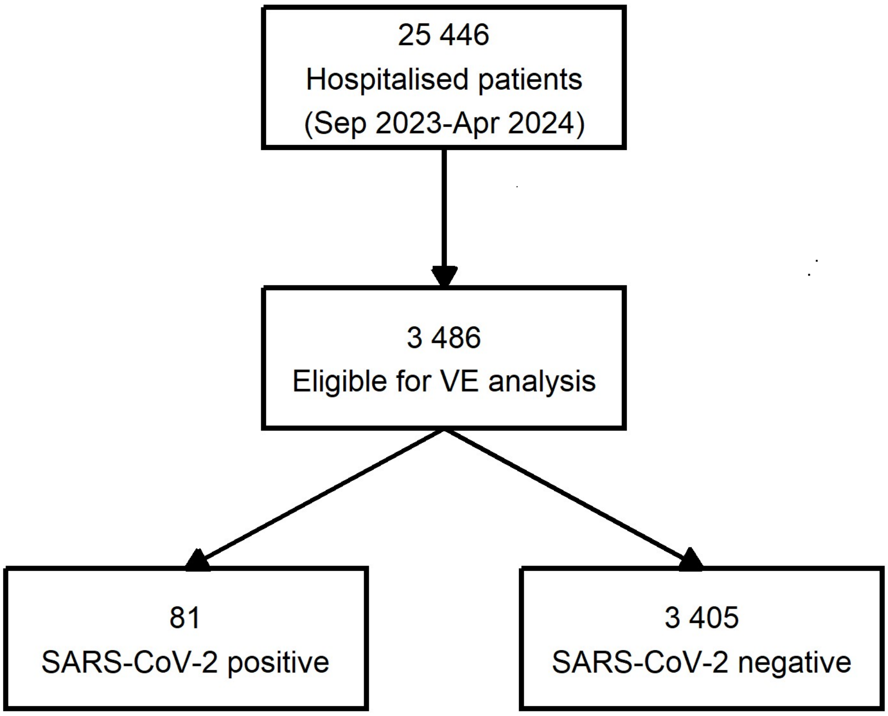

21 960 patients excluded

1 821 outside the analysis period

(1 416 with date of symptom onset before the start of the campaign + 14 days and 405 from 1 site with vaccination data issues before 13 November 2023)

3 436 exclusion criteria (protocol)

(19 missing consent information, 855 missing information on SARI case definition, 1 231 did not meet the ECDC SARI case definition and 1 331 had a missing RT-PCR test result/did not have an RT-PCR test)

9 045 in an ineligible population group

(46 healthcare workers, 925 residents in a long-term care facility and 8 074 aged <65 years)

1 199 missing key covariates for analysis

(3 missing swab date, 13 missing admission date, 127 missing symptom onset date, 16 missing age, 9 missing sex, 306 missing information on common chronic diseases, 287 missing vaccination status and 438 missing date of last received vaccine dose)

471 were ineligible due to timing of symptom onset, swab and hospitalisation

(316 swabbed >10 days after symptom onset, 24 swabbed >3 days before symptom onset, 130 swabbed >48 hrs after hospitalisation and 1 swabbed >14 days before hospitalisation)

3 663 with ineligible vaccination status

(193 with last vaccine dose <14 days before symptom onset, 1 with contraindications for vaccination, 69 with last vaccine dose received within 180 days prior to the campaign, 13 not eligible to be vaccinated in PT and IE, 2 not vaccinated with a booster dose during the XBB.1.5 vaccination campaign in PT and IE, 20 vaccinated with bivalent vaccine, 1 vaccinated with vaccine brand other than Comirnaty, Spikevax or Nuvaxovid, 1 received Comirnaty XBB.1.5 vaccine before 31 August 2023 and 3 363 vaccinated ≥ 60 days before onset)

1 489 additional exclusions

(955 before the first sequenced result in each site, 522 missing or positive sequencing for SARS-CoV-2 variants other than BA.2.86 and 12 controls sequenced for SARS-CoV-2 variants)

**21 960 patients excluded**
**836 due to site restrictions**<br>(4 sites with fewer than 5 cases or controls (n=607) and 3 sites with no vaccinated cases and controls (n=229))
**Records included are from 59 hospitals in five sites (Belgium, Germany, Spain, Ireland, Navarre region and Spain)**
