# Supplementary material for: Modelling approaches for estimating vaccine effectiveness of consecutive SARS-CoV-2 variant sublineages in the absence of study-specific genetic sequencing data, VEBIS hospital network, Europe, 2023/24
Source: PLoS One. 2026 Mar 9;21(3):e0343988. doi: 10.1371/journal.pone.0343988 (PMC12970855; doi:10.1371/journal.pone.0343988)
Supplement: S4 Fig — (PDF) [file pone.0343988.s010.pdf]

S4 Fig. Patient exclusion flowchart for the XBB standard predominance period (standard PP) analysis, VEBIS hospital study, Europe.

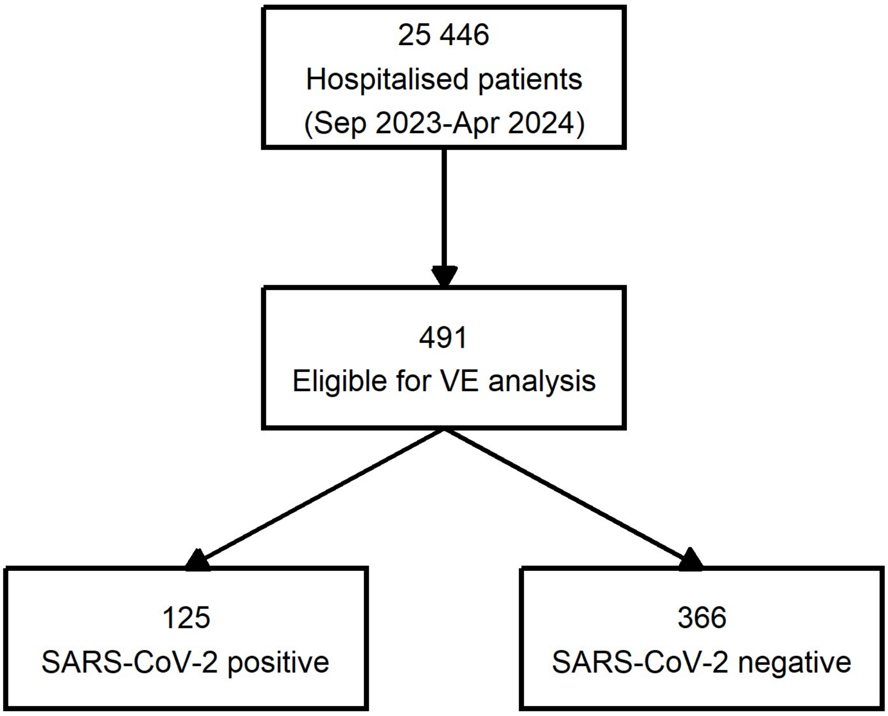

24 955 patients excluded

23 467 outside the analysis period
(1416 with date of symptom onset before the start of the campaign + 14 days, 405 from 1 site with vaccination data issues before 13 November 2023 and 21646 XBB predominant period)

310 exclusion criteria (protocol)
(94 missing information on SARI case definition, 152 did not meet the ECDC SARI case definition and 64 had a missing RT-PCR test result/did not have an RT-PCR test)

741 in an ineligible population group
(5 healthcare workers, 75 residents in a long-term care facility and 661 aged <65 years)

192 missing key covariates for analysis
(2 missing admission date, 18 missing symptom onset date, 2 missing age, 2 missing sex, 25 missing information on common chronic diseases, 25 missing vaccination status and 118 missing date of last received vaccine dose)

55 were ineligible due to timing of symptom onset, swab and hospitalisation
(28 swabbed >10 days after symptom onset, 3 swabbed >3 days before symptom onset, 23 swabbed >48h after hospitalisation and 1 swabbed >14 days before hospitalisation)

90 with ineligible vaccination status
(75 with last vaccine dose <14 days before symptom onset and 15 with last vaccine dose received within 180 prior to the campaign)

100 due to site restrictions
(5 sites with fewer than 5 cases or controls (n=86) and 1 site with no vaccinated cases and controls (n=14))

Records included are from 41 hospitals in four sites (Germany, Spain, Croatia, Navarre region and Spain)
