# Supplementary material for: Modelling approaches for estimating vaccine effectiveness of consecutive SARS-CoV-2 variant sublineages in the absence of study-specific genetic sequencing data, VEBIS hospital network, Europe, 2023/24
Source: PLoS One. 2026 Mar 9;21(3):e0343988. doi: 10.1371/journal.pone.0343988 (PMC12970855; doi:10.1371/journal.pone.0343988)
Supplement: S5 Fig — (PDF) [file pone.0343988.s011.pdf]

S5 Fig. Patient exclusion flowchart for the BA.2.86 standard predominance period (standard PP) analysis, VEBIS hospital study, Europe.

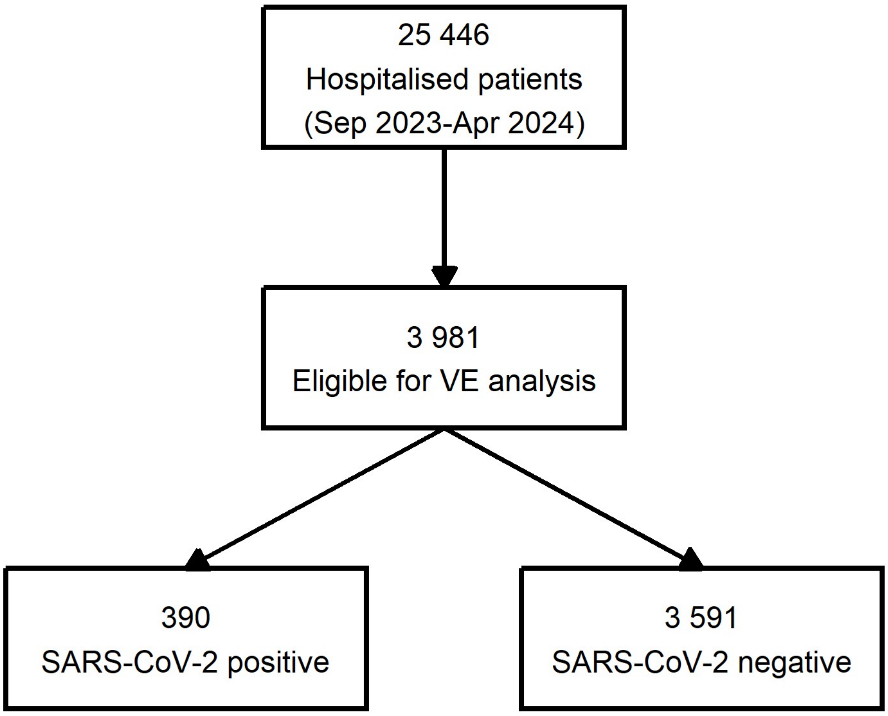

21 465 patients excluded

6 103 outside the analysis period
(1416 with date of symptom onset before the start of the campaign + 14 days, 405 from 1 site with vaccination data issues before 13 November 2023 and 4282 BA.2.86 predominant period)

2 616 exclusion criteria (protocol)
(700 missing information on SARI case definition, 951 did not meet the ECDC SARI case definition and 965 had a missing RT-PCR test result/did not have an RT-PCR test)

7 388 in an ineligible population group
(38 healthcare workers, 737 residents in a long-term care facility and 6613 aged <65 years)

929 missing key covariates for analysis
(11 missing admission date, 106 missing symptom onset date, 13 missing age, 7 missing sex, 270 missing information on common chronic diseases, 239 missing vaccination status and 283 missing date of last received vaccine dose)

374 were ineligible due to timing of symptom onset, swab and hospitalisation
(260 swabbed >10 days after symptom onset, 19 swabbed >3 days before symptom onset and 95 swabbed >48h after hospitalisation)

3 496 with ineligible vaccination status
(58 with last vaccine dose <14 days before symptom onset, 1 with contraindications for vaccination, 47 with last vaccine dose received within 180 days prior to the campaign, 9 not eligible to be vaccinated in PT and IE, 2 not vaccinated with a booster dose during the XBB.1.5 vaccination campaign in PT and IE, 18 vaccinated with bivalent vaccine, 1 vaccinated with vaccine brand other than Comirnaty, Spikevax or Nuvaxovid, 1 received Comirnaty XBB.1.5 vaccine before 31 August 2023 and 3359 vaccinated ≥ 60 days before onset)

559 due to site restrictions

**21 465 patients excluded**
(1 site with fewer than 5 cases or controls (n=166) and 5 sites with no vaccinated cases and controls (n=393))
**Records included are from 60 hospitals in six sites (Belgium, Germany, Spain, Ireland, Malta, Navarre region and Spain)**
