# Supplementary material for: Modelling approaches for estimating vaccine effectiveness of consecutive SARS-CoV-2 variant sublineages in the absence of study-specific genetic sequencing data, VEBIS hospital network, Europe, 2023/24
Source: PLoS One. 2026 Mar 9;21(3):e0343988. doi: 10.1371/journal.pone.0343988 (PMC12970855; doi:10.1371/journal.pone.0343988)
Supplement: S6 Fig — (PDF) [file pone.0343988.s012.pdf]

S6 Fig. Patient exclusion flowchart for the XBB and BA.2.86 adapted predominance period (adapted PP) variant/sublineage category model (VCM) analysis, VEBIS hospital study, Europe.

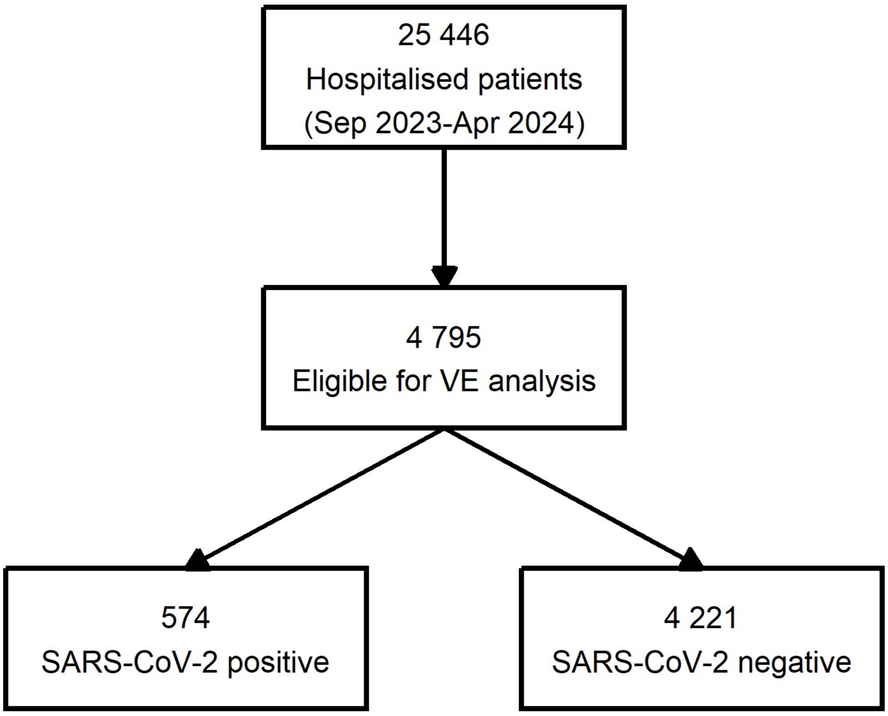

20 651 patients excluded

3 477 outside the analysis period
(1 416 with date of symptom onset before the start of the campaign + 14 days, 405 from 1 site with vaccination data issues before 13 November 2023 and 1 656 between 41% and 59% BA.2.86 circulating)

3 025 exclusion criteria (protocol)
(808 missing information on SARI case definition, 1 145 did not meet the ECDC SARI case definition and 1 072 had a missing RT-PCR test result/did not have an RT-PCR test)

8 402 in an ineligible population group
(43 healthcare workers, 853 residents in a long-term care facility and 7 506 aged <65 years)

1 137 missing key covariates for analysis
(13 missing admission date, 122 missing symptom onset date, 15 missing age, 8 missing sex, 299 missing information on common chronic diseases, 266 missing vaccination status and 414 missing date of last received vaccine dose)

445 were ineligible due to timing of symptom onset, swab and hospitalisation
(299 swabbed >10 days after symptom onset, 23 swabbed >3 days before symptom onset, 122 swabbed >48 hrs after hospitalisation and 1 swabbed >14 days before hospitalisation)

3 614 with ineligible vaccination status
(158 with last vaccine dose <14 days before symptom onset, 1 with contraindications for vaccination, 63 with last vaccine dose received within 180 days prior to the campaign, 11 not eligible to be vaccinated in PT and IE, 2 not vaccinated with a booster dose during the XBB.1.5 vaccination campaign in PT and IE, 18 vaccinated with bivalent vaccine, 1 vaccinated with vaccine brand other than Comirnaty, Spikevax or Nuvaxovid, 1 received Comirnaty XBB.1.5 vaccine before 31 August 2023 and 3 359 vaccinated ≥ 60 days before onset)

551 due to site restrictions

20 651 patients excluded
(1 site with fewer than 5 cases or controls (n=198) and 4 sites with no vaccinated cases and controls (n=353))

Records included are from 62 hospitals in seven sites (Belgium, Germany, Spain, Croatia, Ireland, Malta, Navarre region and Spain)
